# Direct Detection of T- and B-Memory Lymphocytes by ImmunoSpot® Assays Reveals HCMV Exposure that Serum Antibodies Fail to Identify

**DOI:** 10.3390/cells7050045

**Published:** 2018-05-19

**Authors:** Fredrik Terlutter, Richard Caspell, Tobias M. Nowacki, Alexander Lehmann, Ruliang Li, Ting Zhang, Anna Przybyla, Stefanie Kuerten, Paul V. Lehmann

**Affiliations:** 1 Research & Development Department, Cellular Technology Limited, Shaker Heights, OH 44122, USA; fredrik.terlutter@autostereoscopic.de (F.T.); richard.caspell@immunospot.com (R.C.); alexander.lehmann@immunospot.com (A.L.); ruliang.li@immunospot.com (R.L.); ting.zhang@immunospot.com (T.Z.); anna.przybyla@immunospot.com (A.P.); 2 Institute of Anatomy and Cell Biology, Friedrich-Alexander University Erlangen-Nürnberg, Erlangen 91054, Germany; stefanie.kuerten@fau.de; 3 Department of Medicine B, University Hospital of Münster, Albert-Schweitzer-Campus 1, A1, Münster 48149, Germany; t.nowacki@jhwaf.de

**Keywords:** human cytomegalovirus (HCMV), enzyme-linked ImmunoSpot assay, ELISPOT, CD4 T cells, CD8 T cells, B cells, serum antibodies

## Abstract

It is essential to identify donors who have not been infected with human cytomegalovirus (HCMV) in order to avoid transmission of HCMV to recipients of blood transfusions or organ transplants. In the present study, we tested the reliability of seronegativity as an indicator for the lack of HCMV exposure in healthy human blood donors. Eighty-two HCMV seronegative individuals were identified, and their peripheral blood mononuclear cells (PBMC) were tested in ImmunoSpot® assays for the presence of HCMV-specific T- and B-memory lymphocytes. Eighty-two percent (67 of 82) of these HCMV seronegative individuals featured at least one memory cell that was lineage specific for HCMV, with the majority of these subjects possessing CD4+ and CD8+ T cells, as well as B cells, providing three independent lines of evidence for having developed immunity to HCMV. Only 15 of these 82 donors (18%) showed neither T- nor B-cell memory to HCMV, consistent with immunological naïveté to the virus. The data suggest that measurements of serum antibodies frequently fail to reveal HCMV exposure in humans, which may be better identified by direct detection of HCMV-specific memory lymphocytes.

## 1. Introduction

Human cytomegalovirus (HCMV) infects the majority of the human population [1]. The initial HCMV exposure can either occur in the neonatal stage, with the mother infecting the newborn, or later during sexual activity. After an acute phase, the infection typically becomes latent, with the virus persisting asymptomatically in various tissues or peripheral blood mononuclear cells (PBMC). However, in states of immunodeficiency, the infection can reactivate, leading to severe clinical complications [2]. HCMV infection is a common complication not only for organ transplant recipients and for patients undergoing immunosuppressive therapy, but also in states of immunodeficiency associated with infections such as HIV, cancer, or old age [2,3,4,5]. When HCMV reactivates in states of such immunodeficiencies it causes significant morbidity and occasional mortality. Therefore, a major goal in transfusion and transplantation medicine is to identify and select donors who are not infected with HCMV and would thus not infect recipients [6].

The identification of an HCMV-infected status primarily relies on detecting HCMV-specific antibodies in the sera of individuals [6]. The presence of serum antibodies has been considered evidence for previous exposure to infectious agents in general, and HCMV in particular [7], but HCMV serology has been called into question regarding its clinical usefulness for predicting posttransplant HCMV infections [8]. Further, there are contradicting reports on serum antibodies indeed reflecting on cellular immune memory to HCMV [9,10,11], in particular because a role for HCMV reactive T cells has been identified in protecting against reactivation in lung transplant recipients [12]. How reliably do, therefore, serum antibodies reveal exposure of individuals to HCMV?

Antibody molecules in serum have a relatively short half-life, on the order of days to weeks, and therefore their presence in serum depends on ongoing production by B-cell-derived plasma cells [13]. In the course of an immune response, naïve antigen-specific B cells become activated by the antigen, and by antigen-specific CD4 T-helper cells. As a consequence of activation, the B cells differentiate into plasma cells that produce antibodies; at the same time, long-lived memory B cells also emerge [14]. These memory cells can give rise to new generations of plasma cells in the presence of persisting/reappearing antigens and T-cell-help, or in the absence of antigens, long-lived plasma cells can continue to spontaneously secrete antibodies [14]. In either case, the presence of antibodies in serum of individuals results from an active, ongoing antibody synthesis process that may or may not reflect previous antigen exposure. For example, human donors tend to become seronegative over time after vaccinations with tetanus toxoid and diphtheria [15], requiring booster immunizations. In other cases, such as vaccinations with vaccinia virus, antibodies persist lifelong, even if the infectious agent has been cleared decades ago [14]. The biological reason for why antibody production persists in one case but ceases in the other is unknown. In order to determine which of these scenarios applies to HCMV, we investigated whether measuring serum antibodies or direct detection of memory T and/or B cells is more reliable for revealing immunological memory to HCMV. In the present study, we tested 82 donors who were identified as HCMV seronegative and asked the question whether direct detection of T- or B-cell memory to HCMV would match their serodiagnostic results.

## 2. Materials and Methods

### 2.1. Human Subjects and PBMC

All 86 human subjects tested in this study were healthy adults ages 18–77. Donors ID 1, 84, and 86 were seropositive for HCMV, while all other donors (IDs 2–83) scored seronegative for HCMV when tested under Clinical Laboratory Improvement Amendments (CLIA) conditions with the FDA-approved Olympus PK CMV-PA Test System (FUJIREBIO Diagnostics, Inc, Malvern, PA, USA). These PBMC donors were recruited by Hemacare (Van Nuys, CA, USA) and the PBMC were isolated by leukapheresis at Hemacare using Hemacare IRB’s. The PBMC were cryopreserved at CTL (Cleveland, OH, USA), following protocols that maintain full lymphocyte functionality upon thawing [16], and were stored in liquid nitrogen vapor until testing in ImmunoSpot® assays. Thawing, washing, and counting of the cryopreserved cells were done as previously described [17]. Within maximally 2 h after thawing, the cells were transferred into the respective tissue culture conditions required for the different test systems.

### 2.2. CD4 and CD8 Depletion of PBMC

CD4+ and CD8+ T cell subsets were depleted from PBMC using anti-CD4 and anti-CD8 magnetic beads (Stem Cell Technologies, Vancouver, Canada), using a negative selection kit. The depletion assay was performed using manufacturer’s instructions. Briefly, PBMC were incubated with anti-CD4 or anti-CD8 antibodies. Bound CD4 and CD8 cells were incubated with magnetic particles. Using a magnet, CD4 or CD8 cells were retained in the tube and the depleted PBMC population was decanted into a separate tube. The success of cell depletions was established by flow cytometry, typically resulting in 93%–98% reduction of CD4+ or CD8+ cells after CD4 or CD8 cell depletion, respectively.

### 2.3. Antigens

The CEF peptide pool (CEFpp), with peptide antigens from the Cytomegalo, Epstein–Barr, and Influenza viruses (CTL-CEF-002), served as a positive T-cell control [18] (CTL, Cleveland, OH, USA). I-HCMV (inactivated HCMV virions) were purchased as CMV gr. 2 antigens from Microbix (Mississauga, Ontario, Canada). HCMVA (pp65), consisting of a pool of 138 peptides derived from a peptide scan (15-mers with 11 aa overlap) through the 65 kDa phosphoprotein (pp65) of HCMV, were purchased from JPT (Berlin, Germany). CMV pp65(495–503) is an HLA-A2-restricted immune dominant peptide of HCMV [19] and was from CTL. All antigens were dissolved in CTL Test Medium (CTLT-005), which also constituted the negative/medium control.

### 2.4. Human Interferon-γ ImmunoSpot® Assay

The human interferon-γ ImmunoSpot® test kit was from CTL. The assay was performed according to the manufacturer’s recommendations; for a detailed description please refer to the CTL website [20]. In brief, the PVDF membrane was precoated with capture antibody overnight, then washed. The antigens were plated in a volume of 100 μL per well at the specified concentrations. The plates containing the antigens were stored at 37 °C in a humidified CO_2_ incubator until the PBMC were ready for plating. The thawed PBMC were added at 3 × 10^5^/cells per well in 100 μL using wide-bore pipette tips. Plates were gently tapped on each side to ensure even distribution of the cells as they settled and incubated for 24 h at 37 °C in a humidified CO_2_ incubator, except for the cytokine secretion kinetics study. No costimulatory molecules were added to this antigen-stimulation culture. Following completion of spot forming unit (SFU) detection, the plates were air-dried in a laminar flow hood prior to analysis. The plates were scanned and analyzed using an ImmunoSpot® S6 Ultimate Reader from CTL. The numbers of SFU were established with the ImmunoSpot® Software (from CTL), whereby for each antigen stimulation condition vs. the corresponding medium (negative) control, the SmartCount™ and Autogate™ functions were used [21]. In all experiments and for all donors, the negative control wells had less than 10 SFU per well. Spot counts reported for the respective antigen-stimulated test conditions are means and SD from triplicate wells, without the medium control subtracted.

### 2.5. B-Cell ImmunoSpot® Assay

B cells secreting IgG antibodies that are specific for HCMV were detected using a dedicated ImmunoSpot® test kit (CTL-CMV-BF, Cellular Technology Ltd., Cleveland, OH, USA) following the manufacturer’s instructions. As memory B cells do not spontaneously secrete antibody [14], their detection requires polyclonal stimulation for several days, during which memory B cells differentiate into plasma cells, and their subsequent antibody secretion allows them to be detected as antibody-secreting cells (ASC) [22]. For polyclonal stimulation, freshly thawed PBMC were resuspended in CTL-Test B™-media (CTLTB-010, Cellular Technology Ltd., Cleveland, OH, USA) supplemented with polyclonal B-cell stimulator (CTL-BPOLY200) that contains R848 and human IL-2 and is part of the kit. The cells were cultured in an incubator at 37 °C and 5% CO_2_ for 5 days. On the 5th day, cells were counted with CTL’s Live/Dead/Apoptotic cell-counting suite using a CTL S6ULT-00-9000 analyzer, and after washing the cells once, the cells were adjusted to 20 × 10^6^ cells/mL, of which 500,000 cells were plated into 96-well plates that were precoated with Poly-CMV antibody (contained within the kit). The cells were incubated for a period of 24 h at 37 °C, 5% CO_2_ during which the B cells secreted antibody. Thereafter, the plates were decanted and washed three times with 200 µL sterile PBS. The plate-bound IgG SFU were visualized using the anti-IgG detection antibody and precipitating substrate contained in the kit, following the manufacturer’s specifications. For three-color detection of IgG1, IgG2, and IgG3, the antibodies were also from CTL (hB26, hB33, hB40). Following the completion of the B-cell assay, the plates were air-dried in a laminar flow hood prior to being scanned and counted on an ImmunoSpot® S6 Ultimate Reader. The numbers of B-cell SFU were determined using the BasicCount™-Mode of the ImmunoSpot® Software.

### 2.6. Statistical Analysis of ImmunoSpot(R) SFU Counts

ImmunoSpot® counts are normally distributed among replicate wells, permitting the utilization of parametric statistics, i.e., the Student’s *t*-test, for identifying positive responses [21]. Accordingly, the Student’s *t*-test was done comparing SFU in the three antigen-containing replicate wells vs. the SFU counts in the medium control wells. A *p*-value <0.05 was considered as the cut-off for a significant increase, that is, for positivity. However, to be on the conservative side, with borderline results, we made it an additional requirement for positivity that spot counts in antigen-stimulated wells needed to exceed SFU counts in the corresponding medium control by 10 SFU/well.

### 2.7. HCMV PCR Assay

Quantitative CMV DNA PCR was performed using a standardized commercially available COBAS^®^ AmpliPrep/COBAS^®^ TaqMan® CMV Test for fully automated specimen processing, amplification, and detection (Roche Molecular Systems, Mannheim, Germany), which had been calibrated and showed colinearity to the WHO International Standard and had previously been tested for quantitative CMV DNA analysis from isolated PBMCs. Additionally, the cellular genome was quantified by PCR targeting sequences of human aspartoacylase (ASPA, gi|767992172|ref|XR_934026.1) using a Roche Light Cycler FastStart DNA Master Sybr Green 1 Kit (Roche, Germany). Sequences of the forward and reverse primers were aspart-fwd (CCCTGCTACGTTTATCTGATTGAG) and aspart-bwd (CCCACAGGATACTTGGCTATGG). Amplification was performed on a RotorGene (Qiagen, Hombrechtikon, Switzerland). Cycling conditions were as follows: 95 °C, 600 s; 95 °C, 10 s; 62 °C, 10 s; 72 °C, 5 s; 78 °C, 15 s (SYBR measurement) for a total of 45 cycles; 50 °C 30 s. DNA extracts of previously quantified human fibroblasts (4 × 10^5^–4 × 10^3^ genomes/mL) were used as an external standard allowing for the construction of a standard curve and the quantification of cellular genomes in clinical samples. All samples contained 0.6–1.5 × 10^6^ cellular genomes. Half of the HCMV seronegative subjects’ PBMC were subjected to quantitative HCMV DNA PCR analysis. Results are reported as the number of HCMV genome copies/sample or the number of HCMV genome copies/cellular genomes with a calculated detection threshold of 10 copies/5 × 10^5^ cellular genomes.

## 3. Results

### 3.1. Detection of HCMV-Specific CD4 and CD8 Memory T Cells 

As an antigen for detecting HCMV-specific CD4-cells, we selected the inactivated HCMV virus itself (I-HCMV, also referred to in the literature as CMV lysate). Having lost infectivity, such inactivated virus particles essentially constitute extracellular viral proteins for phagocytosis, lysosomal degradation, and subsequent antigen-presentation on MHC class II molecules. As an independent antigen formulation of HCMV-antigens, we tested a pool of 138 15-mer peptides that covered the sequence of the HCMVpp65-protein in steps of 11 amino acids (HCMVpp65). Such peptides can directly bind to the MHC molecules of antigen presenting cells (APC) present in PBMC for recognition by HCMVpp65-specific T cells.

**Figure 1:**
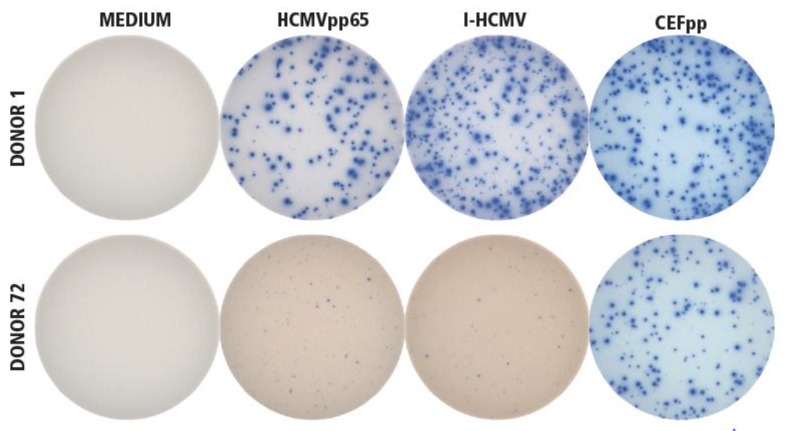
Establishing the specificity of I-HCMV- and HCMVpp65-induced IFN-γ ImmunoSpot® formation. Donor 1 was seropositive for HCMV, Donor 72 was seronegative. PBMC from both donors were tested in an IFN-γ ImmunoSpot® assay in medium alone as the negative control (“MEDIUM”), or in the presence of the HCMVpp65 peptide pool (HCMVpp65) or inactivated HCMV virions (I-HCMV), as specified. As positive controls for eliciting memory T-cell responses, a pool of 32 peptides of HCMV, EBV, and influenza virus were used (CEFpp). The test was performed as specified in Materials and Methods, with three replicate wells for each condition. A representative image of one of the replicates is shown for each condition.

In Figure 1, PBMC of an HCMV-seropositive donor (Donor 1) were tested for the IFN-γ-recall response using an ImmunoSpot® assay. Both antigens, I-HCMV and HCMVpp65, triggered a vigorous IFN-γ response, in addition to the positive control antigen used, the CEF-peptide pool (CEFpp). In contrast, no IFN-γ producing cells were detected when PBMC of an HCMV-seronegative donor (Donor 72) were exposed to I-HCMV and HCMVpp65, but the PBMC of Donor 72 responded to the positive control, CEFpp.

**Figure 2:**
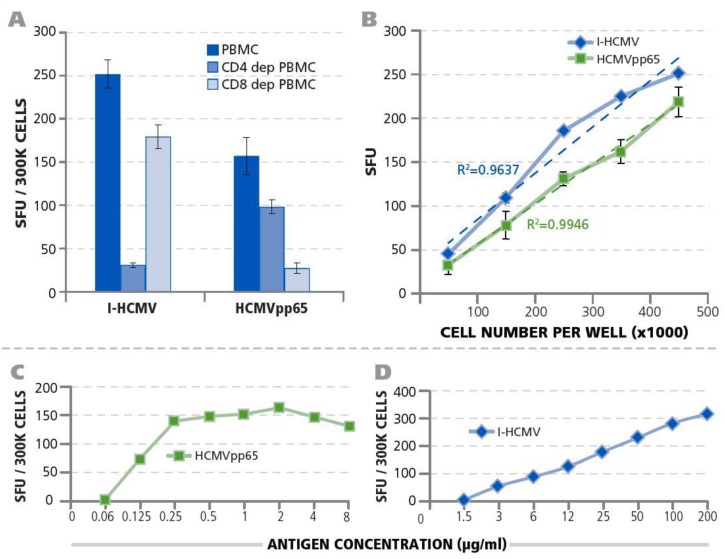
Qualification of HCMV-specific T-cell IFN-γ ImmunoSpot® testing of PBMC. (**A**) Establishing the CD4+/CD8+ lineage of HCMV-specific memory T cells. CD4+ or CD8+ T-cell depleted PBMC or unseparated PBMC of Donor 1 were tested in an IFN-γ ImmunoSpot® assay. The numbers of I-HCMV or HCMVpp65-induced IFN-γ SFU were measured for each condition, and the mean and SD for three replicate measurements of each are shown. (**B**) PBMC cell-number dependence of SFU. PBMC of Donor 1 were plated in the specified cell numbers per well and I-HCMV or HCMVpp65 (specified by color) was added at 50 μg/mL or 1 μg/mL, respectively. The numbers of IFN-γ SFU were established. Means and SD are shown for triplicate wells. The result of regression analysis for the experimental data approaching linearity is specified for both conditions as the *R*^2^ value in color. (**C**) Establishing the optimal antigen dose for stimulation of HCMV-specific T cells by HCMVpp65 peptides. PBMC of Donor 1 were plated at 3 × 10^5^ cells per well along with the different peptide concentrations specified. The number of IFN-γ SFU was measured in single wells. (**D**) Establishing the optimal antigen dose for stimulation of HCMV-specific T cells by I-HCMV. PBMC of Donor 1 were plated at 3 × 10^5^ cells per well along with the different antigen concentrations specified. The number of IFN-γ SFU was measured in single wells.

Next, we identified the cell type within the PBMC that produced IFN-γ after exposure to I-HCMV and the HCMVpp65 peptide pool. We tested unseparated PBMC (which contain CD4+ and CD8+ T cells, as well as APC such as B cells, macrophages, and dendritic cells), along with PBMC fractions that were either depleted of CD4+ T cells or of CD8+ T cells using magnetic-bead selection. As shown in Figure 2A, depletion of CD4+ T cells abrogated the I-HCMV induced IFN-γ-response, whereas the depletion of CD8+ T cells had a minor effect on it. In contrast, the depletion of CD8+ T cells abrogated the HCMVpp65-induced IFN-γ-production, whereas CD4+ T-cell depletion caused a less dramatic decrease. These data establish that the I-HCMV-induced IFN-γ production in PBMC is primarily CD4+ T-cell-derived, whereas the HCMVpp65-triggered IFN-γ was primarily produced by CD8+ T cells.

IFN-γ ImmunoSpot® assays measure the frequency of antigen-specific T cells in PBMC at single-cell resolution [23]. To verify this notion for the I-HCMV and HCMVpp65 antigen systems as well, we tested whether the numbers of PBMC seeded into the assay would correlate linearly with the number of IFN-γ spots (spot forming units, SFU) detected. For both the I-HCMV- and HCMVpp65-induced IFN-γ SFU, a close to perfect linear relation was seen with *R*^2^-values of 0.9637 and 0.9946, respectively (Figure 2B). These data also establish PBMC numbers per well in which frequencies of HCMV-specific T cells can be accurately measured. For subsequent studies, we selected 300,000 PBMC/well.

Further optimizing the assay conditions for detecting HCMV-specific T cells, we titrated both antigens (Figure 2C). For I-HCMV, more than 50 µg/mL of antigen was needed to elicit the maximal number of IFN-γ producing cells, whereas for HCMVpp65 peptide concentrations exceeding 0.5 μg/mL triggered maximal numbers of IFN-γ-producing cells. For the subsequent studies, 50 µg/mL of I-HCMV or 1 μg/mL of HCMVpp65 were used to elicit T-cell responses, respectively.

### 3.2. Detection of HCMV-Specific Memory B Cells 

**Figure 3:**
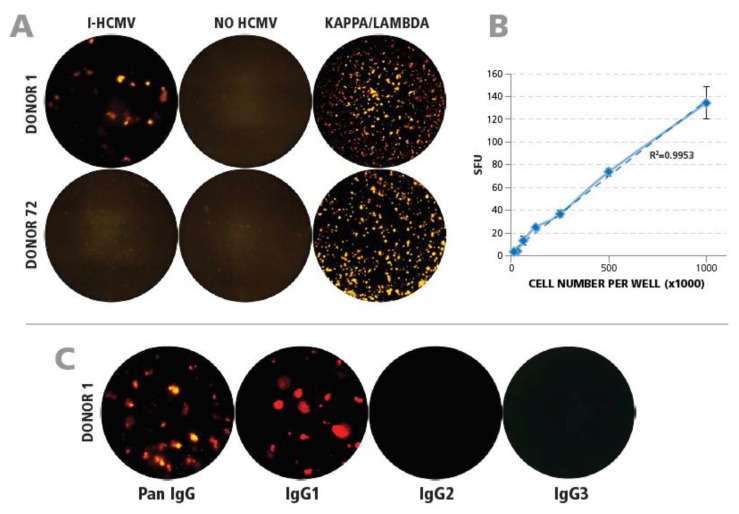
Qualification of detecting HCMV-specific B cells by ImmunoSpot® testing of PBMC. (**A**) Establishing the specificity of the assay. Membranes were coated with either I-HCMV, or with anti-kappa/lambda capture antibody as a positive control, as specified. PBMC of Donor 1 of Donor 72 were added after polyclonal stimulation and plate-bound IgG was detected as described in Materials and Methods. Representative wells are shown. (**B**) Correlation between cell numbers plated and HCMV SFU detected. Preactivated PBMC of Donor 1 were plated in the specified cell numbers and the IgG SFU were counted. The result of regression analysis for the experimental data approaching linearity is specified by the *R*^2^ value. (**C**) Identifying the IgG subclasses of HCMV-specific B cells. Wells were coated with I-HCMV antigen followed by plating of preactivated PBMC of Donor 1. The plate-bound antibodies were visualized using detection antibodies specific for pan-IgG, IgG1, IgG2, and IgG3 as specified.

Resting memory B cells in PBMC do not produce antibodies unless they are first stimulated to become plasmablasts. This can be accomplished by culturing PBMC with R848 and IL-2 for 5 days [22]. Such polyclonally prestimulated PBMC were seeded into ELISPOT assays in which B cells producing HCMV-specific antibodies can be detected. When such prestimulated PBMC of Donor 1 (HCMV-seropositive) were seeded on membranes that were precoated with I-HCMV, plate-bound IgG-antibody spots were detected; in contrast, no such spots were seen in seronegative Donor 72 (Figure 3A). Neither of the two donors produced SFU in assays performed on membranes that were not coated with I-HCMV (Figure 3A). These data establish the HCMV-specificity of the IgG spots detected in the B-cell ImmunoSpot® assay. Using anti-kappa/lambda antibody coating, which detects all B cells that are secreting antibodies, both donors exhibited a strong positive response (Figure 3A). When the prestimulated PBMC of Donor 1 were plated in serial dilution into an HCMV-coated plate, a close to perfectly linear correlation (*R*^2^ = 0.9953) was observed between the number of IgG spots detected and the cell numbers seeded (Figure 3B). As antibody production is confined to B cells, these data establish that the ImmunoSpot® assay used detects precise frequencies of B cells secreting HCMV-specific IgG antibodies.

In follow-up experiments, we defined the IgG subclass(es) that constituted the HCMV-specific IgG antibodies. The B-cell ELISPOT assay was performed as above, by seeding prestimulated PBMC of Donor 1 onto HCMV-coated membranes, but either a detection antibody that binds to all IgG subclasses (pan-IgG) or detection antibodies that selectively bind only IgG1, IgG2, or IgG3 were added. As can be seen in Figure 3C, the frequencies of pan-IgG and IgG1 spots closely matched up while no IgG2 and IgG3 spots were detected. The prevalence of the IgG1 subclass among HCMV-specific IgG antibodies was also seen in 18 additional donors tested (data not shown). For further studies, we relied on detecting pan-IgG.

### 3.3. Testing HCMV Seronegative Human Donors for HCMV-Specific CD4+ T Cells, CD8+ T Cells, and B Cells

**Figure 4:**
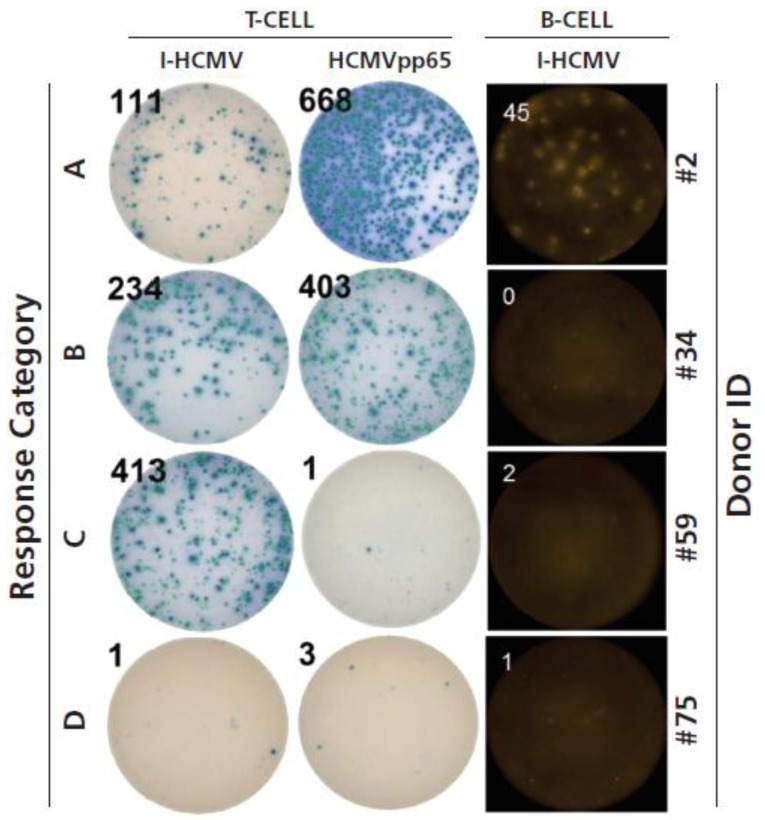
Immune memory response categories defined with representative donors shown. Response Category **A** consists of donors who showed a clearly positive (as defined in Materials and Methods) anti-I-HCMV and anti-HCMVpp65 T-cell response while also displaying memory B cells specific for I-HCMV. Representative wells are shown for Donor 2, who falls into this category. Response Category **B** consisted of donors who displayed I-HCMV- and HCMVpp65-reactive T cells (thus CD4+ and CD8+ memory cells) in elevated frequencies, but who had no detectable HCMV-specific memory B cells. Representative wells are shown for Donor 34, who falls into this category. Donors in Response Category **C** displayed T cells responding to I-HCMV (i.e., CD4+ cells, see Figure 2A) but in the absence of HCMVpp65-reactive T cells and B cells. Representative wells are shown for Donor 59, who falls into this category. Finally, donors of Response Category **D** did not show detectable frequencies of either memory cell type and therefore appeared to be immunologically naïve to HCMV. Representative wells are shown for Donor 75, who falls into this category.

After having established the ideal test conditions for detecting HCMV-specific T cells (both CD4+ and CD8+ cells, each activated by two independent antigen systems) as well as IgG-producing memory B cells, we set out to test PBMC of 82 HCMV-seronegative subjects (Donor ID 2–83). The results are summarized in Table 1, with the PBMC donors grouped according to response categories; examples of such response categories are provided in Figure 4.

Only 15 of the 82 HCMV seronegative human PBMC donors (18%) lacked HCMV-specific T- and B-memory cells (Donor IDs 69–83 in Table 1). These donors’ PBMC responded to third-party antigens such as CPI, however, demonstrating the functionality of these cells (Figure 1 and data not shown). The lack of HCMV responses detected in these donors served as an additional specificity control for the detection of HCMV-specific memory lymphocytes in the ELISPOT assays used, excluding nonspecific (false positive) detection of HCMV memory in Response Categories A, B, and C.

Thirty of the 82 HCMV seronegative donors (37%, Donor IDs 2–28, 46, 50, and 52), could be categorized into Category A, displaying clear-cut immune memory in all three memory-cell lineages. Fourteen donors (17%) were of Category B, displaying T-cell responses to both I-HCMV and HCMVpp65, but low to no B-cell memory to HCMV (Donor IDs 29–40, 48 and 53). Fifteen donors (18%, Donors 54–68) showed CD4+ T-cell responses to I-HCMV in the absence of detectable CD8+ T-cell memory elicited by HCMVpp65, and therefore qualified as Category C. Overall, 67 of 82 (82%) of HCMV seronegative subjects tested (Donor IDs 2–68) displayed at least one, but most of them two or three, independent lines of evidence of possessing memory cells specific for HCMV.

### 3.4. Detection of HCMVpp65 (495-503) Peptide-Specific T Cells in HLA-A*02:01-Positive Donors

**Figure 5:**
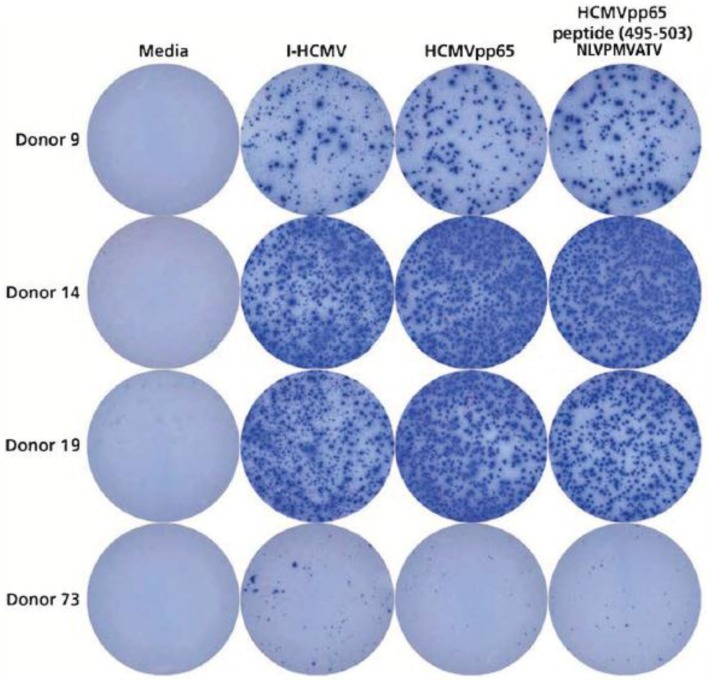
HLA-A*202:01-positive donors, seronegative for HCMV, respond to peptide NLVPMVATV. Four HLA-A*202:01-positive donors were selected who in previous assays were shown to either respond to I-HCMV and the HCMVpp65 peptide pool (Donors 9, 14, and 19), or not to respond to these antigens (Donor 73)—see Table 1. All four donors were retested in an IFN-γ ImmunoSpot® assay for I-HCMV and HCMVpp65 peptide pool reactivity, but this time also testing for the single peptide HCMVpp65(495–503), NLVPMVATV. Each condition was tested in triplicate wells, of which one representative well is shown.

In the above studies aiming at the detection of HCMV-specific T and B cells, we used either HCMV-lysate as the antigen or a sizable peptide pool (HCMVpp65, a pool of 138 15-mer peptides that covered the sequence of the pp65 protein). Because of the complexity of these two HCMV antigen systems, one might argue that they are too crude to assure exquisite specificity for detecting memory cells, possibly leading to false-positive results due to cross-reactivities. To ascertain the HCMV specificity of our T-cell detection system, we extended our study to include a single short synthetic peptide that is known to be immune dominant in HLA-A*02:01-positive individuals, peptide HCMVpp65 (495–503), which has the amino acid sequence NLVPMVATV [24]. Three HCMV seronegative donors have been selected who were HLA-A*02:01 positive and who displayed a high-frequency recall response to I-HCMV and the HCMVpp65 peptide pool (Donors 9, 14, and 19). These donors were tested for reactivity to the NLVPMVATV peptide. Donor 73 was also included in this study as a specificity control, because in spite of also being HLA-A*02:01 positive, this donor neither responded to I-HCMV nor to the HCMVpp65 peptide pool (Table 1). As shown in Figure 5, all three I-HCMV and HCMVpp65 peptide pool reactive donors also responded vigorously to the NLVPMVATV peptide, while Donor 73 did not respond to the NLVPMVATV peptide either. The NLVPMVATV peptide reactivity provides a fourth independent antigenic system to verify the notion that HCMV seronegative individuals can possess high numbers of HCMV-specific memory T cells—in this case, CD8+ cells.

### 3.5. Twenty-Four Hours Antigen Stimulation Is Needed to Detect HCMV-Reactive Memory T Cells. 

**Figure 6:**
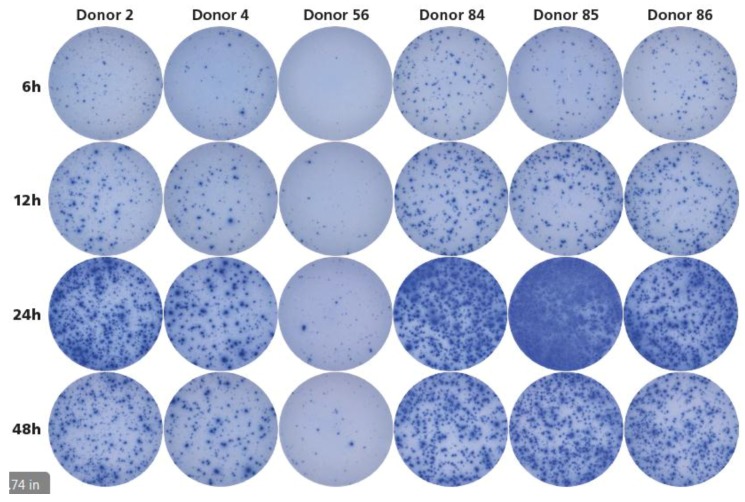
Kinetics of I-HCMV-induced IFN-γ production. Donors 2 and 4 were seronegative for HCMV but were shown to respond to I-HCMV in previous experiments. Donor 56 was also HCMV-seronegative and was shown not to respond to I-HCMV (see Table 1). Donors 84, 85, and 86 were HCMV seropositive. The PBMC of all six donors were retested in an IFN-γ ImmunoSpot® assay at 3 × 10^5^ cells per well after 6, 12, 24, and 48 h antigen stimulation periods with 50 μL/mL of I-HCMV. Each condition was tested in triplicate wells, of which one representative well is shown.

Detection of antigen-specific memory T cells is frequently done by intracytoplasmic cytokine (IFN-γ) staining (ICS), followed by flow cytometric analysis. According to these protocols, the T cells have to be stimulated with antigen for 6 hours before the ICS is performed. Using this ICS-based approach, only 2.1% of the seronegative donors were found to have HCMV-specific memory T cells [10], apparently contradicting our finding reported here that such memory cells are frequently present in HCMV-seronegative donors. The ELISPOT assays we performed to generate the data for Table 1, including a 24 h antigen-stimulation culture, as has been established to be ideal for detecting IFN-γ-secreting T cells in general [25]. By studying the kinetics of the I-HCMV-induced IFN-γ response, we addressed the hypothesis that the underestimation of HCMV-specific T cells by ICS might have resulted from an insufficiently lengthy antigen stimulation period. Two HCMV-seronegative donors were selected who were found to be I-HCMV positive (Donors ID 2 and 4), plus one donor who was I-HCMV negative (Donor ID 56) (see Table 1). In addition, three donors were tested who were HCMV seropositive (Donors ID 84, 85, and 86). The PBMC of these donors were cultured for 6, 12, 24, and 48 h with I-HCMV antigen before the secreted IFN-γ was detected in an ImmunoSpot® assay. As shown in Figure 6, the I-HCMV-triggered IFN-γ production by T cells peaked at 24 h, with it being barely detectable at the 6 h time point. These kinetics were similar for the HCMV-seronegative individuals (Donors 2 and 4), and for the HCMV-seropositive donors (ID 84, 85, and 86). Therefore, measuring cytokine production at the 6 h time point, as commonly done for ICS, does not reveal the full extent of immune memory, as it peaks at 24 h.

## 4. Discussion

Presently, the clinical judgment call whether a human is HCMV positive, and thus can infect an HCMV-negative recipient, is based on serodiagnostic [26]. There are reports, however, that call into question whether HCMV serology testing is indeed clinically useful for predicting posttransplant complications by HCMV infections [8]. Litjens et al. reported [9] that HCMV-specific memory T and B cells can be detected in 46% of HCMV-seronegative patients, and that such HCMV seronegative but memory cell positive subjects had a lower risk to develop HCMV viremia after transplantation with kidneys of HCMV^-^seropositive donors. The authors concluded that it is frequent that humans have HCMV-specific T-cell responses without detectable anti-HCMV antibodies, and that it is clinically relevant, as such memory cells can convey protection for CMV infection.

Apparently contradicting the above notion, Sester et al. [10] reported that of 92 HCMV-seronegative subjects tested, only 2 (2.1%) were found to have increased frequencies of HCMV-specific CD4+ memory T cells as detected by ICS for IFN-γ after stimulation with CMV lysate (which corresponds to the I-HCMV we used here). The lower limit of ICS detection according to the authors was 0.05%. There are several reasons why the presence of T-cell memory to HCMV might have gone undetected in this study. First, we used a more sensitive assay: the detection limit of the IFN-γ ImmunoSpot® assay is substantially lower, at 1:300,000 (0.0003%). Using the latter, we found 67 of 82 HCMV-seronegative donors to be I-HCMV positive (82%, see Table 1). The detection limit of 0.05% for ICS corresponds to 150 SFU/300,000 cells in ImmunoSpot® analysis. Even when taking 150 SFU per 300,000 PBMC as the cut-off, we found 41 of the 82 HCMV-seronegative donors (50%) to be I-HCMV positive (such highly I-HCMV-positive data points are highlighted with dark green in Table 1). Therefore, the higher sensitivity of ELISPOT detection vs. ICS alone does not fully resolve this discrepancy but diminishes it by half. Sester et al. performed ICS after 6 h stimulation with CMV lysate (I-HCMV). According to our findings shown in Figure 6, and similar results communicated earlier [25], the 6 h time point is too short for detecting the activation of all antigen-specific T cells. Accordingly, the 6 h stimulation period used by Sester et al. might have been too short to reveal the full frequency of HCMV-specific CD4+ memory T cells, which, in combination with the 0.05% detection limit, would readily explain why responses went undetected in that study. Overall, therefore, the apparent discrepancy between Sester et al. reporting 2.1% seronegative/T-cell memory positive donors while we finding it to be 82% can be reconciled by the far more sensitive and optimized assay system we used to detect HCMV-specific memory T cells.

Our finding, that a high number of seronegative individuals display immune memory to HCMV is more in line with the findings of Litjens et al. [9], and also of Zhu et al. [27]. The latter authors studied 50 healthy donors, of whom 54% were negative for HCMV serum antibody. Forty-four percent of these seronegative donors displayed a proliferative T-cell response to CMV lysate (that is I-HCMV), whereas, in our study 82% were I-HCMV positive when measured by the ImmunoSpot® assay. The reason why proliferation assays might underestimate the numbers of individuals who have developed an immune response to HCMV by approximately half is also likely to be related to the assay system used. Due to chronic stimulation by the persisting infection—HCMV tends to persist—CMV-specific T cells are prone to undergo replicative senescence, losing their proliferative potential [28]. Terminal effector T cells also lose their ability for self-renewal, and hence go undetected in proliferation assays, but these T cells maintain their ability to produce IFN-γ [29], and thus can be detected by ELISPOT. Proliferation assays, therefore, can be expected to be less sensitive for detecting HCMV-specific T-cell memory than measurements of IFN-γ secretion by ELISPOT, providing the likely explanation to why we have detected twice as many T memory cell positive individuals among HCMV-seronegative subjects.

The above studies aiming to detect HCMV-specific T cells [10,27] used CMV-lysate (I-HCMV) as the antigen, and one could rightfully argue that such an antigen might be too crude to assure a high specificity for the T-cell assays, possibly leading to false-positive results. In addition, HCMV lysate consists of proteins that primarily stimulate CD4+ T cells (Figure 2a, [30]). To ascertain the HCMV specificity of our ELISPOT-based T-cell detection system, we tested synthetic peptides in addition to using HCMV lysate. Testing a peptide pool that covers the entire HCMVpp65 protein sequence systematically with 15-mer peptides that walk the sequence of the protein in steps of 11 amino acids (the HCMVpp65 peptide pool) provided an independent antigenic system for verifying the HCMV specificity of the T-cell response. In contrast to I-HCMV that primarily elicit CD4+ cells, these peptides elicit both CD4+ and CD8+ T cells (Figure 2A). HCMVpp65-antigen-specific T cells were reported to constitute about 12% of the total CMV-specific CD4+ and CD8+ T-cell repertoire, of which HCMVpp65 (495–503) peptide (NLVPMVATV) specific CD8+ cells prevail in HLA-A*02:01 individuals [24]. As a third independent line of evidence for the presence of HCMV-specific T cells in seronegative individuals, we detected CD8+ T-cell memory to NLVPMVATV peptide in HLA-A*02:01-positive subjects (Figure 5). PBMC of some individuals did not display T-cell reactivity to these antigens (Donor IDs 69–83 in Table 1), providing an additional specificity control for the detection of memory cells, and essentially all donors who responded to the HCMVpp65 peptide pool also responded to I-HCMV (see Table 1).

Our detection of HCMV-specific memory B cells in approximately half of the T-cell positive subjects (Table 1) also argues for specific immune memory to HCMV itself as opposed to its cross-reactive recognition. This is because B cells recognize conformational determinants of antigens and T cells recognize peptides that are realigned on HLA molecules. Thus, T and B cells recognize antigens based on fundamentally different criteria, a system that has evolved to minimize the chance for the same antigen being cross-reactively recognized by T and B cells [31].

An important implication of our data is that HCMV-seronegative subjects are more frequently exposed to or infected with HCMV than previously assumed, based on their seronegative status. Presently, to our knowledge, there has been no systematic study published addressing the infectious risk posed by HCMV seronegative but HCMV memory cell positive individuals. The detection of HCMV-specific memory cells does not necessarily imply potential infectivity. We did not detect HCMV genome in PBMC of seronegative/memory cell positive subjects (T.N., data not shown), suggesting that the infectious risk they pose might not be high, but this risk cannot be excluded. On the other hand, the infectious risk might also be mitigated by the fact that a majority of the seronegative recipients, according to the data presented here, have preexisting adaptive immunity to HCMV. Extensive clinical studies will be required to clarify the link between HCMV memory cell status of seronegative individuals, and their infectivity or susceptibility to re/superinfection. Presently, all transplant recipients are given anti-HCMV treatment prophylactically, but there are no precise criteria for when to stop this therapy. Assessing cellular immunity to HCMV by ImmunoSpot® analysis may help to make that decision.

The presence of T-cell immunity in the absence of serum antibodies was first detected in individuals who were exposed to but apparently were not infected by HIV [32,33,34]. Zhu et al. reported similarly discordant cellular and humoral immune responses to HCMV [27], which our data not only substantiate but show to be rather common. Memory B cells reactive to donor HLA molecules have been detected by ELISPOT/Fluorospot in transplant recipients in the absence of antidonor antibodies providing yet another example for the dissociation of cellular and humoral immunity. In the same study, the clinical relevance of this finding was established by showing that these memory B cells confer a higher risk for posttransplant injury [35].

As the half-life of antibody molecules in serum is around 20 days [36], seronegativity implies the lack of plasma cells actively secreting antibodies. In the case of HCMV, we could detect increased frequencies of HCMV-specific B cells that, upon in vitro stimulation, produced IgG antibodies. The former implies clonal expansion, the latter immunoglobulin class switching, both resulting from a T-cell-dependent B cell response [14]. Whether plasma cells secreting antibodies have been induced in these individuals earlier in their life, e.g., upon infection with HCMV, but have stopped producing antibody since is unclear. It is also presently unknown whether after HCMV exposure/infection, like after HIV exposure/infection, the dissociation between cellular and humoral immune memory reflects on successful clearance of the virus, and therefore a lack of infectivity [32,33,34]. To answer this question, on one hand, it will need to be established which cellular and humoral test system is most sensitive and reliable to identify cellular immune memory to HCMV. ImmunoSpot® detection of IFN-γ secreting memory T cells seems so far to be more sensitive than ICS or measurements of proliferative responses. On the other hand, it will need to be established how one can reliably detect dormant virus genomes in organs not readily accessible to PCR analysis to prove that a subject is indeed negative for the HCMV genome, and thus noninfected/noninfectious even though he/she has been exposed to the virus and developed an immune response to it.

HCMV infects many tissues of the body where it can stay latent for prolonged time periods. In latency, the direct detection of the virus is a challenge [37]. As both the organ donor’s and the recipient’s HCMV status is defining the infectious risk associated with transplantation, defining the HCMV serostatus is required before transplantation [6]. Limitations of serologic tests, however, are well known to the field. For example, the interpretation of serology results can be impossible for sera which are obtained after transfusion of blood or blood products due to the transfer of donor antibodies, or in children less than 12 months of age [38]. Our data reported here extend the limitation of serodiagnosis by showing that HCMV-specific B- and/or T-memory cells are present in 82% of healthy seronegative adult donors. These donors have clearly undergone an immune exposure to HCMV, most likely an infection, yet that is not revealed by serum antibody measurements.

The HCMV serology status of the test subjects we studied was established in a CLIA-certified laboratory using the Olympus PK particle agglutination assay, an FDA approved test, commonly performed in blood centers. Clearly, the majority of donors who tested negative in this test when performed in a CLIA-certified laboratory showed evidence of T- and B-cell memory to HCMV. It seemed possible to us that the serum of such donors contained HCMV-specific antibodies that, however, did not induce the agglutination reaction used as the read-out for the Olympus PK assay but might be detected by other means including HCMV ELISA. We therefore retested the sera of 20 OlympusPK negative donors in an HCMV ELISA. Confirming the Olympus PK test results, 18 sera were found negative and 2 were borderline (data not shown). Clearly, serodiagnosis as performed today does not reliably detect HCMV exposure. It will need to be established whether other means of serum antibody detection are more reliable or whether the direct detection of memory B and T cells as shown here is the most reliable way of identifying HCMV exposure, and thus potential infectious risk. Our data call for thorough follow-up studies to this extent.

Given the crucial role of T cells in controlling viral infections in general, and for HCMV in particular [39], attempts have been made to use the detection of activated HCMV-specific CD8+ effector T cells as an indicator of HCMV reactivation. This approach builds on the notion that long-term memory CD8+ T cells do not store Perforin and Granzyme B molecules required for the killing of target cells in their cytoplasm [40,41], whereas recently in vivo reactivated CD8+ T cells do [42,43]. Accordingly, it has been shown that the detection of instantaneous ex vivo Perforin and Granzyme B production by antigen-stimulated CD8+ T cells is suited to detect ongoing T-cell activation in vivo during active viral infections [44,45], and such measurements were also found to hold promise for detecting HCMV reactivation [46]. Granzyme B and Perforin measurements, in addition to IFN-γ detection, while interrogating HCMV-specific CD8+ T cells should facilitate the detection of an active ongoing immune response to the virus, including the detection of a reactivation of HCMV infection.

While the HCMV virus may be sequestered in peripheral tissues during the state of latency, HCMV-specific memory T and B cells will follow their immunological assignment to patrol the entire organism. Memory lymphocytes are known to utilize the bloodstream for their dissemination in the organism and for recirculation among organs [42]. Subsequently, memory T cells can be readily detected in the blood even when the antigen (in this case, HCMV) is sequestered in a tissue such as the gut mucosa. As soon as viral replication stops, these lymphocytes become quiescent memory cells and persist for decades, possibly for the lifetime of the human host. Our approach relied on the detection of such memory lymphocytes belonging to all three major lineages, CD4+, CD8+, and B cells.

Detecting clonally expanded memory T and B cells in seronegative individuals, as we report here, therefore proves that an immune response to HCMV has occurred in these subjects sometime in the past, but does not provide insights on whether the immune system is still actively involved in an encounter with the virus at the time of testing. It also does not permit us to conclude whether such subjects continue to harbor the virus and thus actually constitute an infectious risk. The possibility that these individuals become seronegative because they have cleared the virus, while their memory cells persist, is viable. Our data only proves that these seronegative subjects are not immunologically naïve to HCMV, as had been originally assumed, but have encountered the virus.

Seronegative individuals with CD8+ T-cell memory to HCMV have very likely undergone an HCMV infection. This is because the presentation of an antigen on MHC Class I molecules for CD8+ T-cell recognition in vivo requires active biosynthesis of proteins within the antigen-presenting cells, that is, the engagement of a CD8+ T-cell response in vivo depends on viral replication in infected cells [47]. As the HCMVpp65 peptide pool primarily detects CD8+ T cells (Figure 2), and as at least half of our HCMV seronegative test subjects displayed a vigorous recall response to the HCMVpp65 pool (Table 1), these donors are likely to have undergone HCMV infection. This is clearly the case for the HLA-A*02:01-positive donors who responded vigorously to peptide NLVPMVATV. Studies of HCMV-specific T-cell memory using peptide libraries of 9–10 amino acid length will help elucidate this question because such short peptides bind to HLA Class I molecules only and hence activate CD8+ T cells only [48].

In 15 of 82 seronegative donors (18%) I-HCMV recalled memory T cells, but the HCMVpp65 peptide pool did not (Figure 4C, Donors 54–68 in Table 1). There are two possible interpretations for this finding. First, these individuals might have had an environmental exposure to HCMV antigens without becoming infected. While CD8+ cell induction requires active virus replication, induction of CD4+ T cells does not. Noninfectious virus particles will, therefore, stimulate the induction of a CD4+ cell response without engaging CD8+ cells. In this case, such subjects would be noninfectious. There is a second possible reason for the absence of a response to the HCMVpp65 peptide pool in individuals who respond to the entire virion, I –HCMV. While HCMVpp65 is thought to be an immune-dominant antigen of the virus [14,49], it is not its only immunogenic antigen. Actually, by testing peptide pools that cover 20 open reading frames of HCMV, we found that most of them were targeted by T cells, and that the pp65 protein was only one of those recognized [50]. Thus, it is possible that I-HCMV-positive but HCMVpp65-negative individuals responded to other HCMV antigens than HCMVpp65. Testing such extended antigen libraries, readily feasible by the ImmunoSpot® approach, also provides additional specificity controls for the T-cell diagnosis of HCMV.

In addition to causing clinical complications in states of immunodeficiency, HCMV infection has recently been implicated in the pathogenesis of several autoimmune diseases [51], including rheumatoid arthritis [52] and inflammatory bowel disease [53], as well as vasculopathies and atherosclerosis [54]. Such associations between HCMV and other diseases are mostly based on correlations with HCMV seropositivity. If, however, the majority of seronegative individuals possess memory T cells specific for HCMV, in that case this virus’ contribution to immune pathology might be underestimated. The common pathological mechanism for HCMV contributing to primarily unrelated diseases is that the presentation of HCMV antigens leads to their recognition by HCMV-specific T cells resulting in inflammation in the affected organs. T-cell-mediated inflammation, however, has different qualities depending on the effector lineage of the T cells [55]. A high interindividual variability of HCMV-specific CD4 effector lineages has been described [30] possibly explaining why HCMV antigen persistence, and chronic T-cell responses to the persisting virus, can lead to clinical complications in certain individuals, but not in others, or why even different types of complications are triggered by HCMV-specific T-cell subpopulations. In light of this report, studies of HCMV-associated diseases should involve T-cell diagnosis rather than an assessment of serology, ideally with CD4+ effector lineage determination [30]. As this study shows, serology is not only prone to miss the detection of HCMV-specific T-cell immunity in the majority of seronegative individuals, but also does not reveal the different types of effector lineages/functions with which the HCMV-specific T-cell populations can contribute to immune pathology.

In conclusion, our data suggest a more complex HCMV immunobiology as previously anticipated. The data imply that from a clinical perspective, serologic testing might not suffice for HCMV risk stratification purposes. Our data suggest that measuring HCMV-specific cellular immune memory is a more reliable tool for detecting HCMV exposure of a subject than the measurement of serum antibodies on which the assessment of the HCMV-infected status has primarily relied so far. Using this memory-cell-based approach, many more individuals were found to having been infected by HCMV than was ascertained so far based on serum antibody measurements. In industrialized nations, the number of uninfected individuals was thought to be around 30%. Based on our data, this figure has to be lowered to 18% of these 30%, that is, to as low as 5% (the 15 donors of all 82 seronegative donors we tested, who do not possess detectable levels of either HCMV specific CD4+, CD8+, or B cells). From the transplantation/transfusion perspective, it will be critical to establish in follow-up studies, whether seronegative individuals who show immune memory to HCMV pose an infectious risk. In particular, it will be important to distinguish between individuals who possess HCMV-specific CD8+ memory T cells and thus are likely to have undergone an infection vs. those who are CD8+ T-cell reactivity negative and might only have had environmental exposure to HCMV antigens.

## Figures and Tables

**Table 1:** Establishing the frequency of HCMV-specific T- and B-memory cells in seronegative human donors. For testing T cells, the PBMC of the donors specified by serial numbers were plated at 3 × 10^5^ cells per well, with HCMVpp65 peptide pool at μg/mL, or I-HCMV at 50 μg/mL, as specified. Each condition was tested in triplicate wells, and the mean IFN-γ SFU numbers are shown. The medium control for all donors was less than 10 SFU per well (not shown). Statistical analysis was performed comparing the SFU counts in the three medium control wells with the three replicate wells for HCMVpp65 or I-HCMV. Test results that reached statistical significance (as defined in Materials and Methods) are highlighted in green. Test results exceeding 150 SFU/well, and therefore above the detection limit of flow cytometry, are highlighted in dark green. For B-cell testing, plates were coated with 50 μg/mL I-HCMV and 5 × 10^5^ preactivated PBMC were plated per well. The mean pan-IgG SFU numbers of three replicate wells are specified for each donor. The Donor Serial IDs were assigned after sorting them into the four response categories specified in Figure 4.

Donor Serial #	T-cells		B-cells	Donor Serial #	T-cells		B-cells
	HCMVpp65	I-HCMV	I-HCMV		HCMVpp65	I-HCMV	I-HCMV
1	122	362	45	42	133	515	not perf.
				43	353	661	not perf.
2	668	111	45	44	847	721	not perf.
3	189	77	101	45	281	890	not perf.
4	403	52	21	46	31	20	25
5	29	465	20	47	not perf.	>1000	26
6	243	440	85	48	196	48	9
7	345	459	59	49	2	23	21
8	515	>1000	76	50	27	16	152
9	280	237	38	51	2	73	11
10	607	646	62	52	86	17	17
11	385	764	33	53	21	82	1
12	357	708	13	54	5	104	2
13	401	480	13	55	3	169	1
14	835	>1000	133	56	1	71	1
15	192	435	29	57	5	148	1
16	72	522	23	58	1	74	3
17	551	382	95	59	1	413	2
18	162	385	72	60.	0	83	not perf.
19	792	697	43	61	5	79	not perf.
20	237	336	133	62	3	19	2
21	506	611	89	63	0	46	not perf.
22	533	473	128	64	1	49	not perf.
23	567	433	78	65	0	40	1
24	515	422	48	66	0	26	2
25	23	79	104	67	0	46	1
26	266	193	110	68	1	23	1
27	95	194	72	69	2	4	2
28	465	153	79	70	2	3	1
29	145	202	8	71	2	4	0
30	65	239	8	72	2	4	2
31	298	461	6	73	2	8	3
32	271	519	3	74	2	8	3
33	163	50	4	75	3	1	1
34	403	234	0	76	3	1	6
35	68	148	2	77	5	4	not perf.
36	301	420	2	78	5	4	not perf.
37	176	87	1	79	3	9	not perf.
38	88	433	4	80	1	8	not perf.
39	90	110	5	81	1	3	not perf.
40	52	766	3	82	0	0	not perf.
41	69	239	not perf.	83	1	1	not perf.
